# Differences in Sleep Duration among Four Different Population Groups of Older Adults in South Africa

**DOI:** 10.3390/ijerph14050502

**Published:** 2017-05-09

**Authors:** Karl Peltzer

**Affiliations:** 1 HIV/AIDS/STIs and TB (HAST), Human Sciences Research Council, Pretoria 0001, South Africa; kpeltzer@hsrc.ac.za; Tel.: +27-(0)12-302-2000; 2 Department of Research and Innovation, University of Limpopo, Sovenga 0727, South Africa

**Keywords:** sleep duration, population group differences, older adults, South Africa, Study of Global Ageing and Adult Health

## Abstract

The study aims to investigate sleep duration in four different population groups in a national probability sample of older South Africans who participated in the Study of Global Ageing and Adult Health (SAGE) Wave 1. A national population-based cross-sectional study with a sample of 3284 aged 50 years or older in South Africa was conducted in 2008. The questionnaire included socio-demographic characteristics, health variables, and self-reported sleep duration. Results indicate that White Africans compared to other population groups had the lowest mean sleep duration (7.88 h among men and 7.46 h among women). The prevalence of short sleep was the highest among both men and women among the White African (18.8% in men and 16.9% in women) and Indian or Asian African population groups (14.5% in men and 17.1% in women), and lowest among both men and women in the Black African (7.0% in men and 6.5% in women) and multi-ancestry population groups (15.6% in men and 12.7% in women). The prevalence of long sleep was among both men and women the highest in the Black African population group (56.2% in men and 58.5% in women), and the lowest in the White African population group (36.4% in men and 24.3% in women). In a Poisson regression model, adjusted for sociodemographics and chronic disease status, coming from the male and female White African population group was associated with short sleep. In addition, coming from the Indian or Asian African population group was associated with short sleep. No population group differences were found regarding long sleep prevalence. White Africans reported more short sleep duration than the other population groups, while there were no racial or ethnic differences in long sleep. White Africans are more likely to have sleep durations that are associated with negative health outcomes. An explanation of the high short sleep prevalence among White Africans may be related to their racial or ethnic minority status in South Africa.

## 1. Introduction

In several studies, older age has been found associated with shorter sleep duration [1,2,3]. The National Sleep Foundation recommended 7 to 8 h sleep for older adults, considering better mental and physical health outcomes [4]. Short sleep duration (6 h or less) has been associated with a number of negative health outcomes, including cardiovascular diseases, diabetes mellitus, obesity, and mortality [5,6,7]. There may be gender differences in the outcome of short sleep, as studies found significant associations between short sleep and diabetes mellitus among men and not women, but vice versa for hypertension and stroke [5].

Sleep duration has been found to differ by racial or ethnic group [7,8]. Ethnic or racial minority status has been identified in several studies [9,10] as a predictor of shorter self-reported sleep durations (6 h or less). For example, in U.S. American studies, including among older adults, the prevalence of short (6 h or less) and/or long sleep duration (9 h or more) has been found to be higher in African-Americans, Latinos, Chinese Americans, Asian Americans, and African/Caribbean immigrants compared to European Americans [7,8,11,12,13,14,15,16,17]. In a study on ethnic differences in sleep duration in Amsterdam, The Netherlands, the prevalence of short sleep was higher in all studied ethnic minority groups (Surinamese, Ghanaian, Turkish, and Moroccan) than in the ethnic Dutch, while there were no ethnic differences in long sleep [9]. 

Insufficient sleep has been shown to be associated with cardiovascular disease, obesity, and diabetes among Blacks [6,15]. The greater prevalence of short sleep in specific racial or ethnic groups such as Blacks may suggest that sleep-based approaches to improving cardiometabolic and other health outcomes may need a more multidimensional approach that includes adequate sleep in this population [12].

There are no studies, to my knowledge, investigating ethnic or racial differences in sleep duration in Africa, even though short sleep duration is a common sleep disturbance in Africa [18]. Short sleep is a potential symptom of a sleep disorder (e.g., insomnia) but is not considered an actual sleep disorder. Sleep disorders that can cause short sleep duration may include untreated sleep apnea.

Ethnic or racial differences in sleep duration have been reported in high income countries. It is possible that such ethnic or racial differences may differ by gender [19], and there may be gender differences in the negative health outcome of short sleep [5]. It is therefore important to explore gender differences in sleep duration. It is not known if these ethnic or racial differences persist in low- or middle-income countries such as South Africa. If no differences or opposite differences in sleep duration are found among Black South Africans, this may point to factors, suggesting that the short sleep duration in Black Americans reported above could be related to their minority status.

The study aims to investigate differences in sleep duration among four different population groups (Black Africans, multi-ancestry: Coloured, Indian or Asian African, and White African) in a national probability sample of older South Africans who participated in the Study of Global Ageing and Adults Health (SAGE) in 2008. It is hypothesized that sleep duration differs by ethnic, racial, or population groups in South Africa.

## 2. Materials and Methods

### 2.1. Sample and Procedure

A national population-based cross-sectional study with a sample of 3840 person aged 50 years or older in South Africa was conducted in 2008. The SAGE sample design entails a two-stage probability sample that yields national and sub-national estimates to an acceptable precision at provincial level, by locality type (urban and rural), and by population group (including Black African, multi-ancestry (Coloured), Indian or Asian African, and White African); more details [20]. At the household level, all individuals aged 50 years and above were invited to participate in the interview; proxy respondents were identified for selected individuals who were unable to complete the interview [20].

The individual response rate among those aged 50 years or older was 77%. The Global Study on Ageing (SAGE) survey was carried out in South Africa in partnership between the World Health Organization (WHO), the National Department of Health, and the Human Sciences Research Council (HSRC) [20]. The study was approved by the Human Sciences Research Council Research Ethics Committee (Protocol REC 5/13/04/06) and the national Department of Health, and written informed consent was obtained from participants.

### 2.2. Measures

#### 2.2.1. Sleep Duration 

The survey included two questions about self-reported hours of sleep last night and the night before last: “How many hours (and minutes) did you sleep?” This was a closed question in which interviewees had to report the number of hours and minutes. The sleep duration of the two days was averaged. In order to capture possible non-linear associations between sleep duration and its associates, we followed a similar categorization used by previous studies and classified sleep hours into ≤6–8, and ≥9 h per day [8].

#### 2.2.2. Population Groups 

Participants were asked, “What is your background or racial group?” The response options were “Black African, Coloured, Indian/Asian, White and other”; those that responded “other” were excluded from this analysis. According to Statistics South Africa ([21], p. 12) “in South Africa, analysis by the four population groups remains an important crosscutting variable to better understand and plan for education, employment, health, mortality, fertility, and migration within the country.”

#### 2.2.3. Economic or Wealth Status

To estimate economic or wealth status, a hierarchical ordered probit model was utilized in order to develop an index of household asset ownership of durable goods (such as chairs, tables, cars, television, telephone, and washing machine), dwelling characteristics (such as type of floors, walls, and cooking stove), access to services such as improved water and sanitation, electricity, and type of cooking fuel used in the household [22]. This enabled an estimation of an asset ladder. These estimates of thresholds, combined with actual assets observed to be owned for any given household, were used to produce an estimate of household-level wealth status. This was the used to create wealth quintiles [19].

#### 2.2.4. Educational Level 

Educational level was assessed by asking participants, “How many years of school, including higher education, have you completed?” The response option was the number of years.

#### 2.2.5. Urban and Rural Residence

Urban and rural residence was assessed based on the survey methodology according to the classification of studied enumeration areas based on population census data [20].

#### 2.2.6. Chronic Conditions 

Chronic conditions such as arthritis, stroke, angina, asthma, chronic lung disease, and diabetes were assessed by self-report (coded 1 = having any of the six conditions, 0 = no condition).

#### 2.2.7. Sociodemographic Variables

Age, gender, educational level and residence.

### 2.3. Data Analysis

Using STATA software version 13.0 (Stata Corporation, College Station, TX, USA), data were analyzed taking the sampling design. To test for differences among population groups and urban–rural residence χ^2^ tests, analyses of variance (ANOVAs) with posthoc comparisons and Student *t*-tests were used. Associations between confounding variables (socio-demographics and having chronic conditions) [8,12,16,23] and short and long sleep prevalence were evaluated calculating prevalence ratios (PR) using Poisson regression. In the analysis, weighted percentages are reported. The reported sample size reflects the sample that was asked the target question. The *p*-value of less than 5% was used to indicate statistical significance. Both the *p*-value and the reported 95% confidence intervals are adjusted for the multistage stratified cluster sample design of the study.

## 3. Results

### 3.1. Sample Characeristics

The total sample included 3284 of 50 years or older South Africans, 44.1% men and 55.9% women. The most prevalent population group was Black African (74.0%), followed by multi-ancestry (12.8%), White African (9.3%), and 3.8% Indian or Asian African. Compared to other population groups, the Black African population group had the lowest educational level, the lowest wealth, and lived more likely in a rural area. The multi-ancestry and Indian or Asian African population group seemed to have a higher prevalence of having any chronic condition than the Black African and White African population groups (see Table 1).

### 3.2. Sleep Duration

Table 2 shows the mean sleep duration and the prevalence of short and long sleep in men and women and in participants residing in urban and rural areas by population group. Among men, the average sleep duration reported was shorter in White Africans (7.88 h) compared to the other population groups (ranging from 8.00 h among Indian or Asian Africans to 8.72 h among Black Africans). Likewise, among women, the mean sleep duration was significantly higher in all population groups (ranging from 7.85 h from among Indian or Asian Africans to 8.96 h among Black Africans) than in the White African population group (7.46 h). Among all four population groups, except for White Africans, the mean sleep duration was significantly higher in participants residing in rural areas than those residing in urban areas.

The prevalence of short sleep was the highest among both men and women among the White African (18.8% in men and 16.9% in women) and Indian or Asian African population groups (14.5% in men and 17.1% in women), and lowest among both men and women in the Black African (7.0% in men and 6.5% in women) and multi-ancestry population groups (15.6% in men and 12.7% in women). The prevalence of long sleep was among both men and women the highest in the Black African population group (56.2% in men and 58.5% in women), and the lowest in the White African population group (36.4% in men and 24.3% in women). Except for the Black African and White African population groups, in the other two population groups the prevalence of short sleep was higher among those residing in urban than those in rural areas. Except for the White African population group, in the other three population groups, the prevalence of long sleep was higher in older adults residing in rural than urban areas (see Table 2).

### 3.3. Prevalence Ratios for Short and Long Sleep by Population Group

In Model 2, White Africans were more likely than Black Africans to report shorter sleep durations even after accounting for age, gender, education, wealth status, residence, and chronic disease. In addition, coming from the Indian or Asian African population group was associated with short sleep. In Model 1, adjusted for age, White African woman and Indian or Asian African women were less likely to be long sleepers than Black African women were. However, in Model 2—adjusted for age, education, wealth, residence and chronic disease status—no population group differences were found in both men and women regarding long sleep prevalence (see Table 3).

## 4. Discussion

This study examined differences in sleep duration among Black African, White African, multi-ancestry, and Asian Africans in a representative sample of older South Africans. Previous research focused on investigating these variables predominantly in high-income countries (North America and Europe) and found evidence that the prevalence of short and long sleep duration was higher in ethnic minority groups such as Blacks compared to European Americans [7,8,11,12,13,14,15,16,17]. This study found a reverse result that the prevalence of short sleep (6 h or less) was for both men and women the highest among White Africans (a minority group in South Africa) compared to all other population groups (dominant and other minority groups), even after controlling for residence. On the other hand, the prevalence of long sleep (9 h or more) was among both men and women the highest among Black Africans compared to all the other population groups; however, this became non-significant after adjusting for confounders. One contributing factor to this was that the prevalence of long sleep was in three population groups higher in those residing in rural compared to urban areas, and a large proportion of the Black Africans resided in rural areas, while the vast majority of the White Africans resided in urban areas. Other studies [24] also found that when neighbourhood disparities are adjusted for racial or ethnic disparities, sleep duration might be minimized.

Reasons for the high prevalence of short sleep found in the White African population group are not clear and may be attributed to other factors than sociodemographic, economic, and health indicators. If one would apply the migration stress hypothesis [9], apart from Black Africans, White Africans, Indian or Asian Africans migrated at some stage to South Africa, and the multi-ancestry group is mixed between Black Africans and predominantly Europeans. Accordingly, the study found the highest prevalence of short sleep among White African and Indian or Asian African population groups, followed by multi-ancestry and the lowest prevalence of short sleep among Black Africans. Further, it is possible that when humans left Africa, genetic differences between different ethnic or racial groups were acquired that caused differences in sleep rhythms in White Africans and Indian or Asian Africans prior to their return to South Africa [7].

Another concern found in this study was the high prevalence of long sleep duration among Black Africans and multi-ancestry women. “Considerable evidence shows that long sleep duration (≥9–10 h) in older adults is associated with morbidity (e.g., hypertension, diabetes, atrial fibrillation, poor general health) and mortality. Excessive sleep may be a marker in older adults signalling the need for medical, neurological, or psychiatric evaluation” ([4], p. 236).

Sleep health intervention may be required to specifically address sleep health disparities among White Africans and Indian or Asians Africans in South Africa [25]. Sleep behaviour may be a more easily modifiable lifestyle factor than dietary or physical activity behaviour, and may therefore have significant potential to improve cardio metabolic health [7].

This study had several limitations. Firstly, the self-report of health variables such as sleep duration and chronic conditions. For example, compared with objective methods, self-reported sleep duration tends to over report sleep, which is at a higher rate among short sleepers than long sleepers [26]. This could mean that, in this study, a larger proportion of short sleepers would have been found with the use of objective sleep measures. This study was a cross-sectional survey, so no causality can be claimed. Further, the sample size for some of the population groups studied was small, thus generating large confidence intervals. Future studies should have a sufficiently large sample size for each population group. Furthermore, the study did not assess whether the night of sleep reported was weekend vs. weekday, and as the sleep duration may differ, this is a potential limitation of the study.

## 5. Conclusions

This study showed that short sleep duration differed by population group, with the highest prevalence of short sleep among White Africans and Indian or Asian Africans, followed by multi-ancestry and the lowest prevalence of short sleep among Black Africans. Such ethnic or racial differences were not found for long sleep duration. This information may help in sleep health planning and programming. Further, research should investigate how these ethnic or racial differences in short sleep may be related to ethnic differences in physical and mental health, and may contribute to health disparities such as cardiovascular disease.

## Figures and Tables

**Table 1:** Sample characteristics by population group and gender (*N* = 3284).

Variable	Black African	White African	Multi-Ancestry	Indian/Asian African	*p*-Value
**Men**	*n* = 803	*n* = 132	*n* = 232	*n* = 136	
	M (SD)	M (SD)	M (SD)	M (SD)	
Age	62.1 (9.5)	63.1 (10.0)	62.3 (8.6)	62.2 (8.6)	0.714
	*n* (%)	*n* (%)	*n* (%)	*n* (%)	
Education					<0.001
0–6 years	485 (61.1)	5 (1.1)	112 (45.6)	31 (15.7)	
7–11 years	200 (27.1)	50 (36.5)	93 (40.6)	66 (42.1)	
12 or more years	87 (11.8)	67 (62.4)	14 (13.8)	34 (42.3)	
Wealth					<0.001
Low	429 (52.2)	4 (4.3)	78 (21.8)	11 (22.7)	
Medium	132 (16.2)	8 (4.3)	49 (9.2)	22 (12.8)	
High	236 (31.6)	119 (91.5)	104 (69.0)	103 (64.5)	
Residence					<0.001
Urban	432 (59.4 )	104 (84.2)	179 (87.1)	127 (80.2)	
Rural	371 (40.6)	28 (15.8)	53 (12.9)	9 (19.8)	
Chronic condition					<0.001
No	488 (62.3)	66 (60.7)	117 (41.0)	53 (38.4)	
Yes	285 (37.7)	58 (39.3)	114 (59.0)	76 (61.6)	
**Women**	*n* = 1250	*n* = 137	*n* = 423	*n* = 171	
	M (SD)	M (SD)	M (SD)	M (SD)	0.098
Age	63.2 (10.2)	64.0 (9.1)	62.9 (9.7)	61.4 (8.1)	
	*n* (%)	*n* (%)	*n* (%)	*n* (%)	
Education					<0.001
0–6 years	756 (60.1)	7 (5.9)	206 (40.1)	80 (46.2)	
7–11 years	336 (30.6)	62 (39.7)	172 (46.3)	68 (34.3)	
12 or more years	108 (9.3)	64 (54.4)	20 (13.6)	17 (19.5)	
Wealth					<0.001
Low	626 (49.5)	4 (2.0)	134 (26.5)	12 (6.9)	
Medium	284 (24.2)	6 (4.3)	116 (23.6)	31 (16.2)	
High	334 (26.3)	126 (93.8)	173 (49.9)	127 (76.9)	
Residence					<0.001
Urban	699 (54.5)	114 (85.5)	366 (91.0)	158 (90.0)	
Rural	549 (45.5)	23 (14.5)	57 (9.0)	13 (10.0)	
Chronic condition					<0.001
No	559 (44.3)	61 (47.5)	148 (30.4)	58 (33.3)	
Yes	647 (55.7)	69 (52.3)	270 (69.6)	99 (66.7)	

M: mean; SD: standard deviation.

**Table 2:** Descriptives of sleep duration by population group.

Variable	Black African		White African		Multi-Ancestry		Indian/Asian African	
**Gender**	**Men (*n* = 803)**	**Women (*n* = 1250)**	**Men (*n* = 132)**	**Women (*n* = 137)**	**Men (*n* = 232)**	**Women (*n* = 423)**	**Men (*n* = 136)**	**Women (*n* = 171)**
	M (SD)	M (SD)	M (SD)	M (SD)	M (SD)	M (SD)	M (SD)	M (SD)
Mean sleep ^1^ (hour)	8.72 (2.3) _a_	8.96 (2.1) _A_	7.88 (1.5) _b_	7.46 (1.8) _B_	8.35 (1.9) _a_	8.27 (2.0) _B_	8.00 (2.0) _b_	7.85 (2.1) _B_
	*n* (%)	*n* (%)	*n* (%)	*n* (%)	*n* (%)	*n* (%)	*n* (%)	*n* (%)
Short sleep ^2^	57 (7.0) _b_	64 (6.5) _B_	24 (18.8) _a_	25 (16.9) _A_	21 (15.6) _b_	49 (12.7) _B_	20 (14.5) _a_	32 (17.1) _A_
Long sleep ^2^	441 (56.2) _a_	754 (58.5) _A_	42 (36.4) _b_	37 (24.3) _B_	99 (44.6) _b_	187 (38.2) _B_	55 (46.7) _b_	54 (36.6) _B_
**Residence**	Rural (*n* = 920)	Urban (*n* = 1131)	Rural (*n* = 51)	Urban (*n* = 218)	Rural (*n* = 110)	Urban (*n* = 545)	Rural (*n* = 22)	Urban (*n* = 285)
	M (SD)	M (SD)	M (SD)	M (SD)	M (SD)	M (SD)	M (SD)	M (SD)
Mean sleep ^3^ (hour)	8.97 (2.2) _a_	8.76 (2.1) _b_	7.93 (1.6) _a_	7.60 (1.7) _a_	8.86 (2.0) _a_	8.19 (2.0) _b_	9.09 (1.9) _a_	7.83 (2.0) _b_
	*n* (%)	*n* (%)	*n* (%)	*n* (%)	*n* (%)	*n* (%)	*n* (%)	*n* (%)
Short sleep ^4^	57 (8.0) _a_	63 (5.7) _b_	5 (17.9) _a_	44 (17.8) _a_	4 (2.5) _a_	66 (15.1) _b_	0 (0.0) _a_	52 (18.7) _b_
Long sleep ^4^	573 (61.1) _a_	621 (54.8) _b_	16 (11.4) _a_	63 (33.2) _b_	58 (58.8) _a_	228 (38.4) _b_	12 (72.6) _a_	97 (35.4) _b_

^1^ Means with differing subscripts within rows by population group are significantly different at the *p* < 0.05 based on Fisher’s least significant difference (LSD) post hoc paired comparisons, _a_ for men and _A_ for women; ^2^ Differences in proportion with differing subscripts across population groups based on χ^2^ statistics at *p* < 0.05, _b_ for men and _B_ for women; ^3^ Means with differing subscribes within rows by urban–rural are significantly different at the *p* < 0.05 based on Student’s *t*-tests; ^4^ Differences in proportion with differing subscripts by urban–rural based on χ^2^ statistics at *p* < 0.05.

**Table 3:** Prevalence ratios for short and long sleep among population groups and gender.

Variable	Black African	White African	*p*-Value	Multi-Ancestry		Indian/Asian African	*p*-Value
		**PR (95% CI)**		**PR (95% CI)**		**PR (95% CI)**	
**Men**							
**Short sleep**							
Model 1	1 (Reference) ^1^	2.82 (1.51, 5.27)	0.002	2.23 (1.21, 4.11)	0.012	2.03 (0.86, 4.82)	0.104
Model 2	1 (Reference)	2.08 (1.13, 3.84)	0.020	1.65 (0.92, 2.96)	0.092	1.66 (0.97, 2.85)	0.063
**Women**							
**Short sleep**							
Model 1	1 (Reference)	2.83 (1.59, 5.04)	<0.001	1.95 (0.95, 4.07)	0.067	2.89 (1.44, 5.81)	0.004
Model 2	1 (Reference)	2.62 (1.44, 4.73)	0.002	1.93 (0.88, 4.24)	0.099	2.72 (1.35, 5.49)	0.006
**Men**							
**Long sleep**							
Model 1	1 (Reference)	0.62 (0.33, 1.16)	0.132	0.78 (0.52, 1.16)	0.213	0.82 (0.52, 1.30)	0.307
Model 2	1 (Reference)	0.82 (0.45, 1.48)	0.499	0.82 (0.55, 1.24)	0.346	0.83 (0.64, 1.39)	0.388
**Women**							
**Long sleep**							
Model 1	1 (Reference)	0.40 (0.19, 0.84)	0.018	0.65 (0.40, 1.05)	0.077	0.60 (0.44, 0.83)	0.002
Model 2	1 (Reference)	0.51 (0.23, 1.13)	0.093	0.68 (0.43, 1.06)	0.088	0.73 (0.51, 1.05)	0.089

Model 1: adjusted for age; ^1^ Reference category is the Black African population group; PR = Prevalence Ratios; CI = Confidence Interval; Model 2: adjusted for age, education, wealth, residence, and chronic condition.
